# Over-expression, purification and isotopic labeling of a tag-less human glucose-dependent insulinotropic polypeptide (hGIP)

**DOI:** 10.1007/s13205-013-0181-x

**Published:** 2013-10-31

**Authors:** Rakesh C. Chandarana, Vikrant, Ashok K. Varma, Anil Saran, Evans C. Coutinho, Jacinta S. D’Souza

**Affiliations:** 1 Department of Pharmaceutical Chemistry, Bombay College of Pharmacy, Kalina, Santacruz (E), Mumbai, 400098 India; 2 Structural and Molecular Biology Laboratory, Tata Memorial Centre, Advanced Centre for Treatment, Research and Education in Cancer, Kharghar, Navi Mumbai, 410 210 India; 3 UM-DAE Centre for Excellence in Basic Sciences, Kalina Campus, Santacruz (E), Mumbai, 400098 India

**Keywords:** Glucose-dependent insulinotropic polypeptide, Affinity chromatography, Isotopic labeling, Recombinant fusion protein, Factor Xa protease cleavage site, Diabetes mellitus

## Abstract

Glucose-dependent insulinotropic polypeptide (GIP), a gut peptide released in response to food intake brings about secretion of insulin in a glucose-dependent manner upon binding to its receptor, GIPR. GIP–GIPR has emerged as a new vista for anti-diabetic drug discovery and their interaction is being probed at the atomic level to aid rational drug design. In order to probe this interaction on cells, the current study attempts towards expressing ^15^N-labeled GIP using classical molecular biology tools. We have developed a methodology to obtain GIP devoid of extra amino acid(s); a prerequisite to the intended interaction study. The synthetic GIP cDNA with a Factor Xa protease site at the N-terminus of GIP was inserted in the vector pET32a(+); the fusion protein thus expressed was eventually cleaved to obtain GIP. After successful Factor Xa cleavage, the cleaved GIP was confirmed by western blot. Subsequently, the (^15^N)GIP was obtained using the aforementioned procedure and confirmed by MALDI-TOF.

## Introduction

Glucose-dependent insulinotropic polypeptide (GIP) is a peptide hormone released into the blood stream in response to glucose and nutrients absorption from the intestine along with glucagon like polypeptide-1 (GLP-1) (Drucker 2006; McIntosh et al. 2009). Both peptides are known to exert varied physiological actions on different body tissues with the pancreas being the major effector. These peptides interact with their respective G-protein-coupled receptors present on the cell surface and activate the adenylate cyclase signaling pathway. It brings about insulin secretion from the pancreas in a glucose-dependent manner and thus the name GIP. This pathway of insulin secretion is known as entero-insular axis and the peptides as incretins. Due to the glucose-dependent action, both GIP and GLP-1 have attracted immense attention for the design of novel anti-diabetics. In order to identify the key determinants of their interaction, attempts have been made to study their structures (Parthier et al. 2007; Runge et al. 2008; Underwood et al. 2010). In particular, studies aimed at probing the interaction of the peptides with the N-terminus domain of their receptors is of interest. Currently, nuclear magnetic resonance (NMR) is the only technique that permits determination of the structure of biomolecules at atomic-level resolution in near-physiological conditions (solution state) (Billeter et al. 2008; Banci et al. 2010). With isotopic labeling, determining complex structures and studying the interaction dynamics of biomolecules in the presence of other biomolecules and within the cell is now feasible (Stockman 2002; Takeuchi and Wagner 2006; Banci et al. 2010). However, much of the success of structural biology depends on the availability of biomolecules in its utmost pure and native state. Moreover, techniques such as X-ray crystallography and NMR require proteins in mM concentration. With the advent of recombinant DNA technology (rDNA) and techniques of gene synthesis, affinity tag-based purification and subsequent removal of the tag by protease cleavage, it is now possible to obtain proteins in their native states with higher yields (Arnau et al. 2006).

Expression of isotopic hGIP had not been reported till date. The current study reports a method of isotopically (^15^N)-labeled and unlabeled recombinant over-expression of hGIP in pET32a(+) vector, with complete tag removal by Factor Xa cleavage and purification of the native hGIP. The peptide obtained here has been characterized by western blotting using hGIP-specific antibody and the labeling has been confirmed by MALDI.

## Materials and methods

All the materials were obtained from Sigma-Aldrich India Ltd., Merck-Millipore India, Himedia (India) and were of molecular biology grade.

### Designing the DNA construct for *hgip* gene

To overcome the problem of codon bias and facilitate complete removal of the tags, the cDNA encoding hGIP was commercially gene synthesized (First Base, Singapore) with a nucleotide sequence coding for an Factor Xa protease cleavage at 5′ and a stop codon at the 3′ ends. The cDNA was cloned into pET32a(+) and subsequently transformed into *Escherichia coli* BL21(DE3) and *E. coli* DH5α strains along with appropriate positive and negative controls. The colonies grown in Luria–Bertani (LB) agar plates containing ampicillin was checked for the presence of the insert by colony PCR and the amplicons were electrophoresed on a 2 % agarose gel containing ethidium bromide. Glycerol stocks of the positive clones were prepared and cryopreserved at −80 °C. Plasmids were isolated from the cells containing the construct and sequenced using pET32a(+)-based primers.

### Over-expression and purification of pET-GIP fusion protein

The *E. coli* BL21(DE3) cells harboring the construct were grown to an OD of 0.6 in 10 ml LB medium containing ampicillin and induced using 1 mM IPTG. In order to determine an optimum time of expression, induction was carried out for 1, 3 and 18 h. The cells collected at different time-points were harvested by centrifugation at 5,000*g*/4 °C and the expression of pET-GIP fusion protein was checked by electrophoresing on a denaturing gel. The solubility of the fusion protein was ascertained by disrupting the cell lysate under non-denaturing conditions and electrophoresing the supernatant (~100 μg total protein/lane) on a denaturing gel. The pET-GIP fusion protein was confirmed using an antibody to the hexa-histidine portion of the tag. Upon observing over-expression of the 23 kDa pET-GIP polypeptide on SDS-PAGE, the conditions were scaled up to 1 l (culture volume) for 3 h and the cells harvested by centrifugation at 5,000*g* for 10 min at 4 °C. The cell pellets were stored at −80 °C until the next step of purification. At the time of purification, the pellet was thawed and the cells were re-suspended in lysis buffer (20 mM Tris–HCl buffer pH 7.5, 300 mM NaCl, 1 mM PMSF, 10 % glycerol and 10 mM imidazole) and ruptured by sonication. The supernatant was collected after centrifuging the cell lysate at 10,000*g* for 30 min at 4 °C and mixed with Ni–NTA beads that were pre-washed with the lysis buffer, in a 50-ml centrifuge tube. The tube was kept on a cell mixer and the binding of the fusion protein to the beads was carried out for 2 h at 4 °C. After binding, the slurry was loaded on a BioRad elution column and the flow-through was collected. The column was washed with lysis buffer containing 20 mM imidazole to remove the non-specific binding of proteins to the column. Elution of the pET-GIP fusion protein was then carried out using imidazole gradient from 20 mM to 2 M in the same buffer and the various fractions were electrophoresed on a denaturing gel. The purity of the fusion protein was assessed by silver staining of the gel. Subsequent purification of pET-GIP fusion protein was carried out by the batch elution method where the protein was eluted using 5 ml of lysis buffer containing 100 mM, 500 mM and 1 M imidazole. The fraction containing the protein was dialyzed against the Factor Xa digestion buffer −20 mM Tris–HCl pH 6.5, 50 mM NaCl, 1 mM CaCl_2_ at 4 °C.

### Factor Xa cleavage of pET-GIP fusion protein

The cleavage of the purified pET-GIP fusion protein obtained in the Factor Xa digestion buffer after dialysis was optimized by varying parameters such as temperature and enzyme concentration. The cleaved protein was electrophoresed on a denaturing gel along with the uncleaved control and molecular weight marker and different fragments were observed. The fragments obtained upon cleavage were also characterized by western blot using hGIP-specific antibody to confirm the presence of the peptide.

### Purification of GIP from the mixture by gel filtration

The 5-kDa hGIP was purified at room temperature by separating it from the mixture containing the residual tag (18 kDa), other contaminants (>11 kDa) and Factor Xa enzyme (55 kDa) by gel filtration using a superdex 75 column on a GE AKTA FPLC system in 20 mM Tris–HCl buffer (pH 7) 150 mM NaCl. The elution was monitored by UV detector (280 nm) and elutes were collected as 2 ml fractions. The fractions showing the protein were electrophoresed on 15 % SDS-PAGE and the separation was checked.

### Isotopic (^15^N) labeling and characterization of hGIP by MALDI

Upon optimizing the basic strategies for over-expression, tag removal and purification of hGIP, isotopic labeling of the peptide was carried out by growing the *E. coli* BL21(DE3) cells up to OD_600_ of 0.6 in the minimal medium (M9) containing isotopic (^15^N) ammonium chloride as the nitrogen source and induced using 1 mM IPTG. Subsequent steps of purification were carried out in the manner as reported for unlabeled hGIP.

The isotopic peptide (0.5 μl corresponding to 0.25 μg concentration) was mixed with the non-isotopic hGIP (0.5 μl corresponding to 0.4 μg concentration) in 1 μl of Sinapinic acid as matrix and spotted on a ground steel MALDI-TOF plate (Bruker Daltonics, USA). This mixture was allowed to dry at room temperature and the plate was then loaded into the instrument. Subsequent to adjusting the appropriate parameters, the spot was fired using the linear mode at 22 ± 2 °C, 27 kV and a microchannel plate detector. The molecular weight was determined using an external standard (insulin, ubiquitin i, cytochrome *c*, myoglobin that covered a mass range of ~5,000–17,500 Da) at an error of ±100 ppm, the software for deconvolution being Flex analysis, Bruker Daltonics.

## Results and discussion

**Fig. 1:**
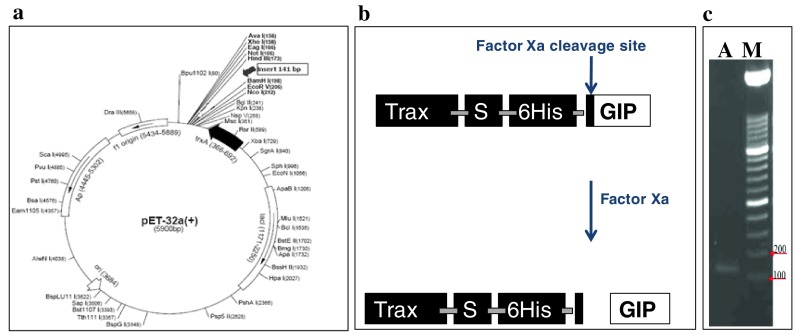
Design of the construct resulting in the cloning of the GIP ORF. **a** pET32a(+) plasmid (obtained from First Base, Singapore) showing the position (between *Bam*HI and *Hin*dIII) at which the h*gip* insert was cloned. **b** A cartoon depiction of the Factor Xa cleavage site. Note that upon cleavage the released hGIP is untagged. **c** PCR of the h*gip* amplicon of ~125 bp; lane labeled *M* is the standard molecular weight marker, lane labeled *A* is the h*gip* amplicon

The need to study the structure of biomolecules at near-physiological conditions is mandatory for rational drug design; hGIP being one of them. For this purpose, cellular and molecular biologists as well as pharmacologists have felt the need to obtain decent concentrations and yields of hGIP in their studies. For long, such experimentalists have been using either the synthetic peptide or purifying it in bulk from natural sources (Pederson and Brown 1976; Alaña et al. 2004). Besides, acquiring (^15^N)hGIP or doubly labeled (^15^N, ^13^C)hGIP synthetically for structural and/or receptor-binding studies has proven to be cost intensive. The aim of the present study was to obtain an economic and constant source of unlabeled and labeled hGIP in reasonable yields using recombinant DNA technology. The pET series of vectors have T7 promoters that over-express proteins in *E. coli* BL21(DE3) cells (Studier and Moffatt 1986). Apart from the hexa-histidine affinity tag, pET32a(+) also has a localization tag (S-tag) and a solubilization tag (ThioredoxinA, TrxA) (Tsunoda et al. 2005). To prevent the possibility of the protein routing into inclusion bodies, the idea of tagging it with a TrxA tag was sought after. The S-tag would aid in future receptor-binding studies under physiological conditions. The current study has directed efforts towards obtaining a constant source of unlabeled as well as labeled hGIP. For the ultimate need of obtaining a tag-less GIP, a construct was designed suitably using pET32a(+) as the backbone vector. The ThioredoxinA (TrxA) tag was followed by the Streptavidin (S) tag, the hexa-histidine (6XHis) tag and a Factor Xa cleavage site (IEGR). The GIP sequence was introduced after this cleavage site so that the tag-less GIP alone is released upon Factor Xa cleavage. The synthetic sequence (Fig. 1a, b) so designed was then synthesized commercially by First Base, Singapore. The resultant recombinant vector (pET32a-*fXa*-*gip*) was used to transform *E. coli* BL21(DE3) competent cells so as to yield colonies on LB agar plates containing ampicillin as the antibiotic of selection. Subsequent characterization by colony PCR using insert-specific and vector-specific primers showed desired amplicons on 2 % agarose gel indicating the presence of the appropriate insert in all the colonies (Fig. 1c). The sequencing of the plasmids isolated from three such positive clones confirmed the presence of the correct sequence of the insert.

### Over-expression and purification of pET-GIP fusion protein

**Fig. 2:**
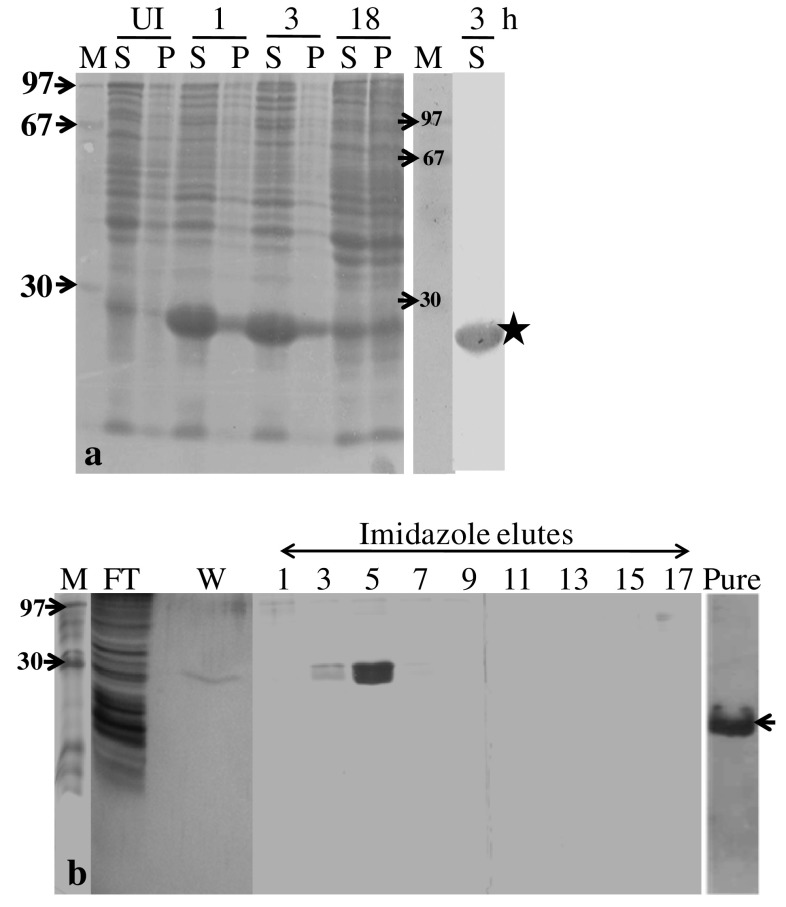
Induction of the fusion protein (Trx-S-6XHis-fXa-GIP). **a** Kinetics of the over-expression of the fusion protein, induction with IPTG and detection of the 6XHis tag using an anti-hexa-histidine antibody (denoted by a *star* in the last lane of this figure). **b** Affinity (IMaC) based purification of the fusion protein using gradient elution (20–2,000 mM imidazole). Note the purification of the fusion protein in elutes 3 and 5. A silver stained gel of the purified protein (see *arrowhead*). Lanes labeled *M* protein molecular weight standards, *P* pellets, *S* supernatants obtained after extraction, *UI* uninduced control, *numbers* time-points (in hours) of induction, *FT* flow-through, *W* wash obtained after the proteins have been bound to the column; numbers against *arrows*, molecular weights in kDa; pure, purified protein (~5 μg)

*Escherichia coli* BL21(DE3) strains containing the plasmid (pET32a-fXa-gip) were induced with 1 mM IPTG. An intense band of fusion protein (Trx-S-6XHis-fXa-GIP) was observed at ~23 kDa; the intensity of this band increased with the duration of induction (Fig. 2a). The optimum time for induction was found to be 3 h, beyond which the protein degraded. As expected, due entirely to the TrxA tag, the fusion protein was found to be soluble as it was released into the aqueous medium by non-denaturing cell disruption (Fig. 2a; S for supernatant fraction that contained the fusion protein and not in the pellet P). The presence of the 6XHis tag, as confirmed using an anti-6XHis antibody on a western blot of the fusion protein (Fig. 2a; last lane) permitted affinity-based purification by IMaC using Ni–NTA matrix.

The purification of pET-hGIP fusion protein carried out using Ni–NTA matrix and an imidazole gradient (20 mM–2 M) showed the presence of pure 23 kDa fusion protein in the elutes when electrophoresed on 15 % SDS-PAGE (Fig. 2b). The protein eluted in the fraction corresponding to ~100–150 mM imidazole and subsequent batch purification of the ^15^N-labeled fusion protein at 100, 250 and 1,000 mM imidazole resulted in elution of maximum protein at 100 mM (Fig. 2b).The silver stained gel showed ~99 % purity and absence of any contaminants. The yield of the fusion protein as estimated by Bradford was found to be ~30 mg/l culture volume.

### Factor Xa cleavage of the fusion protein, purification by gel filtration and MALDI-TOF analysis

**Fig. 3:**
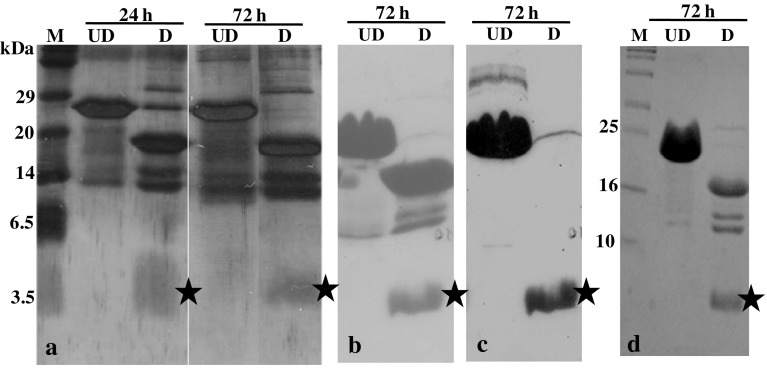
Factor Xa digestion and characterization by western blotting. **a** The fusion protein (75 μg) was digested (lanes labeled *D*) with Factor Xa and the digested products were electrophoresed on a denaturing gel and silver stained; **b** Ponceau S staining of the nitrocellulose membrane after transferring the digested products (starting amount used was 200 μg) by the western blotting procedure; **c** immunoblot for the detection of unlabeled hGIP (starting amount used was 150 μg), and **d** labeled hGIP, using anti-hGIP antibodies. *UD* and *D* labels in all the figures refer to undigested and digested, respectively. The *stars* in all the figures show the position of the digested product, viz. hGIP

After obtaining the purified fusion protein, the conditions for release of the GIP peptide after Factor Xa cleavage were explored. The digestion with Factor Xa was found to be optimum at pH 7.5, 4 °C, enzyme concentration of 1 unit of protease and digestion time being 72 h (Fig. 3a). Upon electrophoreses of the cleaved fragments on a 15 % SDS-PAGE, distinct bands of *M*_r_ ~18 kDa (corresponding to the residual tags) and ~5 kDa (corresponding to hGIP; Fig. 3a) were observed. Another doublet of *M*_r_ ~14 kDa was also seen and possibly is a result of degradation of the fusion protein. In order to ascertain the digested product as hGIP (~5 kDa), a western blot of the digested products was probed with the anti-hGIP antibody (Fig. 3b, c; hGIP is marked with a star). This confirmed that the band was of hGIP and also permitted for further purification of the peptide.

**Fig. 4:**
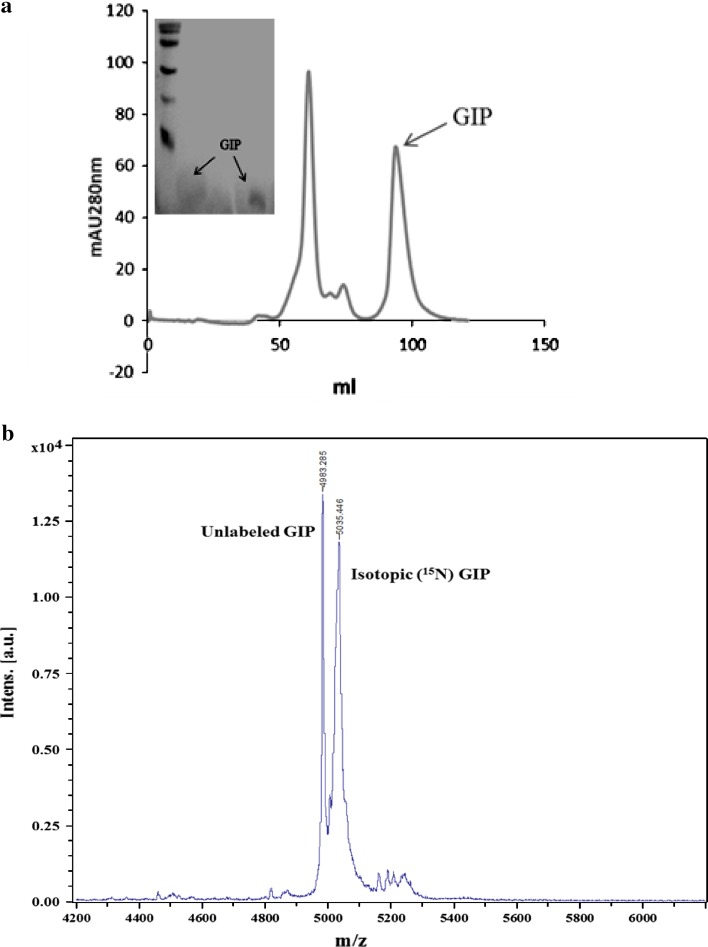
**a** Isotopically labeled fusion protein was digested with Factor Xa and the GIP was purified using gel filtration chromatography on Superdex 75 column on an FPLC system (GE, AKTA). The *inset* is the purified protein that was obtained after digestion. **b** Molecular weight determination of unlabeled and isotopic (^15^N)GIP by MALDI of unlabeled and isotopic (^15^N)GIP

The over-expression of the pET-hGIP fusion protein was reproducible when the cells harboring the construct were cultured in minimal media (M9) containing isotopic (^15^N) ammonium chloride and induced using 1 mM IPTG. Subsequently, the purification of pET-GIP fusion protein was achieved successfully by affinity chromatography on a Ni–NTA column. Although the labeled protein eluted at a higher imidazole concentration (250 mM imidazole), the conditions of digestion with Factor Xa were reproducible. Further purification of hGIP was evident from the chromatogram where different fragments of the digested pET-GIP protein separated differentially by gel filtration on a Superdex 75 column, the tag and the doublet eluted in the 22nd–38th fraction (44–74 ml) and the hGIP at 41st–54th fraction (82–108 ml) showing a peak in the 47th fraction (94 ml). When the fraction corresponding to the hGIP peak was loaded on 15 % SDS-PAGE it showed the hGIP fragment in purified form (inset of Fig. 4a). The peptide was concentrated by ultrafiltration using amicon column mwco 3 kDa. The characterization of the labeled hGIP by MALDI showed the desired peak precisely at 4.98 kDa corresponding to the unlabeled peptide and also a peak at 5.44 kDa corresponding to the molecular weight of the ^15^N labeled peptide. This confirmed the isotopic labeling of the hGIP. The yield of the hGIP peptide was ~1 mg/l of the culture.

It may be noted that GIP (full-length) or truncated versions have thus far been produced synthetically (Fehmann and Göke 1995). Such synthetic peptides have been used for various binding as well as physiological studies. However, these have been useful in proton-NMR studies and thus far no such method has been developed to obtain isotopic (^15^N)hGIP. Hence, this is the first report of ^15^N-labeled hGIP and we present a clone that harbors a fusion gene containing the h*gip* gene that can be reproducibly used to produce the fusion protein at ~30 mg/l and upon digestion and further purification, a peptide of almost 99 % purity with a yield of ~1 mg/l.
